# Perceptual Training in Ice Hockey: Bridging the Eyes-Puck Gap Using Virtual Reality

**DOI:** 10.1186/s40798-025-00840-x

**Published:** 2025-04-12

**Authors:** Jean-Luc Bloechle, Julien Audiffren, Quentin Sauthier, Quentin Mertenat, Yohann Waeber, David Aebischer, Jean-Pierre Bresciani

**Affiliations:** 1 Control and Perception Laboratory, University of Fribourg, Bd Perolles 90, Fribourg, CH-1700 Switzerland; 2 HC Fribourg-Gotteron, Granges-Paccot, CH-1763 Switzerland

**Keywords:** Ice hockey, Perceptual training, Virtual reality, Puck-view, Visual feedback

## Abstract

**Background:**

Some cognitive and perceptual determinants of sports performance can be arduous to train using conventional methods. In ice-hockey, this is the case for the players’ ability to identify the largest exposed area (LEA), i.e., the goal area that is the least covered by the goaltender from a puck perspective. We developed a virtual reality (VR) application to quantify and train the players’ ability to identify the LEA from a wide range of shooting positions. Thirty-four professional ice-hockey players were tested. Between two test sessions, half of the players followed a specific feedback-based training (feedback group), whereas the other players practiced without feedback (control group).

**Results:**

For the players of the feedback group, perceptual performance was significantly better after training, whereas it remained unaltered for the players of the control group. For both groups, perceptual performance decreased as the amplitude of the eyes-puck difference (i.e., the difference of perspective between the eyes and the puck) increased. This relationship vanished after training for the feedback group but not for the control group.

**Conclusions:**

We took advantage of VR technology to assess and train the perceptual ability to identify the LEA from a puck perspective, which would be difficult using traditional methods. Only 15 min of specific feedback-based training significantly and substantially improved the perceptual performance of professional ice-hockey players, thereby evidencing the effectiveness of our application for training an important perceptual skill in ice hockey.

**Supplementary Information:**

The online version contains supplementary material available at 10.1186/s40798-025-00840-x.

## Key Points

This work presents a virtual reality simulator that quantifies and trains the perceptual ability of ice hockey players to identify the largest exposed area when attempting a shot.

The simulator was tested on thirty-four professional players, and a single session of specific training resulted in a 15% improvement of their perceptual performance.

**Supplementary Information:**

The online version contains supplementary material available at 10.1186/s40798-025-00840-x.

## Background

For many decades, research on the determinants of sports performance mostly focused on three main factors, namely physiological and cardiorespiratory fitness [1–7], muscular and anthropometrical / biomechanical characteristics [8–12], and psychological resilience / mental strength, i.e., the ability to cope with stress and maintain high levels of motivation [13–16]. These factors constitute such obvious and undeniable determinants of sports performance that they are actually ‘embedded’ in the Olympic motto “Citius, Altius, Fortius”, i.e., “faster, higher, stronger”. Other factors, such as perceptual and cognitive factors, also importantly impact sports performance [17–23]. This is especially true for team sports, in which athletes can be saturated with perceptual information and must quickly orient / reorient their attention, select the most relevant information (while ignoring the irrelevant information), process this information, and make the most appropriate decision under the current circumstances [24–27]. However, studies specifically investigating the perceptual and cognitive determinants of sports performance are relatively recent when compared to physiological and anthropometrical studies. A plausible reason for this lag probably lies in the complexity inherent to the investigation of perceptual and cognitive factors, especially in representative and thereby complex sports settings. In particular, the complexity and highly dynamic / interactive nature of team-sports situations make it difficult to present realistic, representative stimuli, and to accurately measure athletes’ performance. This is notably true when compared to research carried out in very ‘static’ and controlled settings such as the typical psychology or neuroscience laboratory, where highly controlled and sometimes overly simplified stimuli are used. In recent years, however, the advent and fast development of new technologies, such as virtual reality (VR) and augmented reality (AR), has helped researchers to try to overcome the abovementioned limitations. This has resulted in a growing scientific effort to try to understand which perceptual and cognitive abilities affect sports performance, how to specifically improve these abilities in athletes, and how to develop dedicated virtual environments [28].

VR technology grants the possibility to ‘immerse’ users in rich representative environments that emulate those experienced in real-world settings. In particular, VR grants excellent experimental control [29], so that the stimuli presented to the users can be manipulated or distorted in a perfectly controlled manner in order to suit specific objectives [30]. A straightforward example of such manipulations is the ability to display a different perspective or viewpoint on the visual scene [31–34]. Because it ‘breaks the laws of optics and physics’, this type of manipulation would obviously not be possible using real-world settings. In addition, feedback about performance, be it real-time or delayed, can be effortlessly and flexibly augmented, manipulated or individualized to meet specific needs or goals [33]. This is particularly important because individualized training is crucial for skill learning [35]. All these attributes make VR a very interesting tool to investigate human cognition and behavior [30], as well as to develop training protocols aiming at helping people learn or re-learn motor and cognitive skills [36–42]. This is even more so when considering that VR-based training can provide learning benefits which are similar [43, 44], and in some circumstances superior, to real-world training [45–48]. For all these reasons, VR-based applications are developed in a large number of domains, ranging – in a non-exhaustive way – from the entertainment industry [49–51] to therapy [52–56], telemanipulation [57, 58], training [59–64], or rehabilitation / neuro-rehabilitation [38, 39, 45, 65–67].

The features of VR described above have naturally been exploited to study sports performance and to try to improve athletes’ skills. Specifically, practice is the best and most effective way to improve motor [68–73] as well as procedural skills [60, 74]. By allowing users to interact with representative environments and giving researchers and trainers the possibility to easily control and manipulate these environments, VR constitutes an interesting and entertaining option for sports training [24, 48, 61, 75–87]. An additional important advantage of VR-based training is that it grants more flexible options to increase the difficulty of the task as the level of performance of the user improves [59]. Last but not least, research shows that athletes tend to enjoy VR-based training [88]. Consequently, an always increasing number of VR applications are designed to study and / or improve sports performance [59–64, 84, 89].

We took advantage of the features of VR technology to assess and train the perceptual ability to identify the largest exposed area (LEA), i.e., the goal area that is the least covered/protected by the goaltender when a shot is taken in ice hockey. Specifically, professional ice hockey players were presented with different situations in which they faced the goaltender, and their task was to decide which area was the ‘least covered’ by the goaltender, i.e., where the open area for the puck was the largest. Different approach angles and distances were used. Note that because the puck is relatively far from the shooter’s eyes, the LEA from the eyes perspective is not necessarily the LEA from the puck perspective. This is a very important point because if the eyes of the shooter are his/her window to the world, the relevant perspective when trying to maximize his/her chance of scoring is the puck perspective. Our work specifically addressed this difference of perspective between the eyes and the puck, with two objectives, namely (1) to quantify how well professional players perceptually ‘integrated’ this difference through their many years of practice, and (2) to try to train and improve this perceptual skill to optimize the outcome of shot attempts. The players who participated in the study were randomly assigned to two different groups, and we tested their ‘baseline’ ability to identify the LEA from the puck perspective. The two groups then took a training session during which one group (feedback group) received specific feedback, while the other group (control group) further practiced without feedback. By allowing the learner to compare his/her performance to the ‘ideal’ performance, feedback plays a key role in the learning process, and it has been consistently shown to effectively promote skill acquisition (see [90–93] for reviews). The information that is ‘naturally’ provided by the action is classically defined as intrinsic feedback [71], whereas information supplementing intrinsic feedback is usually defined as augmented feedback [91, 94–96]. Here we capitalized on VR to provide players with a specific type of augmented feedback, namely the possibility to see the visual scene from the puck perspective. Providing this type of feedback would be difficult without resorting to VR or AR technology. The objective was to help players ‘put themselves in the eyes of the puck’. After training, the two groups were re-tested (same test as before training) and the training-evoked improvement of perceptual performance was quantified.

## Methods

### Participants

Thirty-four elite ice-hockey players (all male, aged 18–36, mean = 24.4 ± 5.6) participated in the experiment. Twenty-two of them play left-handed (i.e., holding the stick on the left side of the body with the right hand on top of the stick) and the other twelve play right-handed. All of them play in the Swiss National League (the top-tier of the Swiss league system), and thirty of them also play for the national team of their country of origin. The players were randomly assigned to two different groups, namely the control or the feedback group (17 players per group). Written informed consent was obtained from the participants. The procedures used in the experiment complied with the Declaration of Helsinki for Human Research, and ethical approval was obtained from the Ethics Committee of the University of Fribourg (ethics approval number: 2024 − 925).

### Set-up / Apparatus

For all experimental sessions, participants were seated at a table and wearing a VR headset (Pimax 5K XR). The headset features two QuadHD screens (2560*1440 pixels per eye, 120 Hz refresh rate), so that the visual scene is viewed in stereo with a diagonal field of view of 200 degrees. The headset was connected to a gaming laptop (ASUS ROG Strix SCAR 17.3’’, GeForce RTX 2070 Super graphics, Intel Core i7 CPU, 16GB RAM) via a USB 3.0 cable and a Displayport 1.4. The headset strap and the inter-ocular distance were adjusted for each participant to provide comfort and an optimal viewing experience. A custom-made response box with five response buttons was positioned on the table in front of the participants, mirroring the spatial layout of the ‘target areas’ in the goal (see details below).

The VR simulation / visual scene was developed using the Unity 3D engine and the C# programming language. This approach enabled us to guarantee precise programmatic interactions with the virtual goaltender and adequate feedback throughout trials.

### Visual Scene (Virtual Goaltender)

**Fig. 1:**
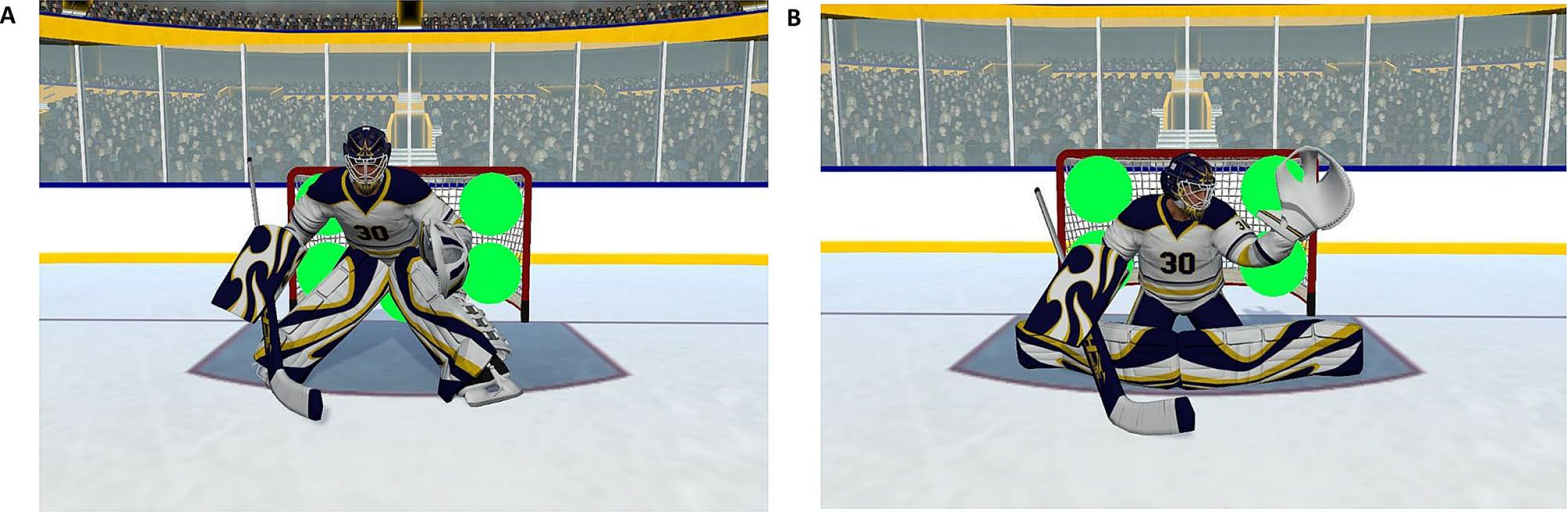
Examples of the player’s view when in shooting position with: **A**. the goaltender in standing position, and **B**. the goaltender in ‘full’ butterfly save position

The visual scene consisted of a virtual ice-hockey rink. Participants were facing one of the goals, which was guarded by a virtual goaltender. When they were in shooting position, five circular targets (0.5 m in diameter) were displayed in green (RGB 0 255 63) in the goal area. Figure 1 shows examples of the player’s view when in shooting position.

The animations of the virtual goaltender were created based on the motion-captured saves of a professional goaltender playing in the Swiss National League. Specifically, the goaltender came to our lab for a two-hour motion capture session. This session was performed on a 3.6 by 2.0 m rectangle of artificial ice (GSI Funice G2 Synthetic Ice), and the goaltender wore his regular hockey outfit. During the session, the goaltender performed various state-of-the-art butterfly saves, executing all movements usually performed by the goaltender on the ice-hockey rink when trying to stop a shot. The goaltender was equipped with 30 infrared reflective markers, arranged into a 6 rigid bodies skeleton marker set, to facilitate precise motion tracking. His movements were captured using 24 infrared cameras (OptiTrack system, NaturalPoint, Inc.) operating at a sampling rate of 120 Hz. Supplemental Fig. 1 illustrates the motion-capture session and the associated animation of the virtual goaltender.

The skeleton and its movements were ‘reconstructed’ by the Motive software (NaturalPoint, Inc.). This reconstruction consisted of creating a skeleton consistent with the morphology of the physical goalkeeper and in updating the skeleton rotations based on the 3D displacements of the markers over time. A 3D mesh of the avatar based on the morphological measurements of the physical goalkeeper was then created. In a post-processing stage, the recorded movements were imported into Unity and the specific save sequences (e.g., butterfly saves) were refined. This process entailed trimming the recorded clips and aligning them within a precise 2-second time frame to ensure accurate timing and fluid movements. Overall, the whole process was very similar to that used to create and animate characters for video games or movies like Avatar. The motion capture session was supervised by a former professional goaltender who played in the NHL and for the Swiss national team and is currently the goaltender coach of a Swiss National League team.

### General Design and Procedures

Before starting the experiment, the players donned their ice-hockey shoes and positioned themselves in their favorite shooting posture. While they were in this posture, the experimenter measured their eye height as well as the position of the puck relative to their eyes (see Supplemental Fig. 2). These data were used to set the camera position (virtual environment) in the first-person and in the puck-view. Put differently, the first-person and the puck-view were adjusted for each player based on the player’s favorite shooting position.

The experiment itself consisted of three sessions, namely pre-test, training, and post-test, which were performed by all players in this chronological order. In the pre-test session, the baseline for perceptual performance was assessed. This session was followed by a training session, in which feedback about perceptual performance was provided to the feedback group, but not to the control group (see details below). For both groups, the post-test session was identical to the pre-test session (except for the order of presentation of the trials, which was random), and it was used to quantify the effect of feedback on perceptual performance in our task. The only difference between the two groups was the training session. Specifically, at the end of each training trial, participants in the feedback group were provided with two types of feedback (see Feedback subsection below) indicating the LEA from the puck perspective. On the other hand, the participants in the control group did not receive any feedback.

### Pre-and Post-Test Sessions

At the beginning of each trial, the player (i.e., the virtual camera) was located at a 5-meter distance from the shooting point, that is at either a 8.5 or 10.5–12.5 m distance from the goal, on the arc of a circle centered at the center of the goal. Three seconds after the beginning of the trial, the camera tracked in along a straight line towards the goal at a speed of 10 km/h, simulating a forward motion of the player. The forward motion stopped at either a 3.5-, 5.5- or 7.5-meter distance from the goal. During the approach/track-in phase, the virtual goaltender either executed a complete butterfly save and dropped on his knees, or initiated the movement but stopped before completing it and therefore remained standing (incomplete butterfly save). For each type of butterfly save (i.e., complete and incomplete), two variations of the recorded saves were used, so that a total of four saves were used during the experiment. The saves of the goaltender were synchronized with the camera’s movement, i.e., they were triggered based on the position of the player. In the pre- and post-test sessions, six different approach angles were used, namely 45°, 25°, 5°, -5°, -25°, and − 45°. For all approach angles, the goaltender faced the player. For instance, at a 5° approach angle, the player started the sequence near the offensive zone’s center, with the goaltender perpendicular to the goal line, ready to face the player’s shot. At the end of each approach sequence, the goaltender was on a semi-circle 1 m in front of his net. When the track-in motion stopped (i.e., after the goaltender had executed his save movement), five target areas lit up light green, prompting the player to select a target using the response box within a 2-second window. Specifically, the player had to select the target corresponding to the LEA, i.e., the area least covered/protected by the goaltender. The player submitted his response by pressing one of the buttons on the response box. The spatial arrangement of the five buttons corresponded to the configuration of the five target areas within the goal (see Supplemental Fig. 3).

The trial was over after a response button was pressed or after the two seconds elapsed without response. For each test session, the players performed 3 trials per shooting location, and the order of presentation of the trials was fully randomized. In total, each test session consisted of 54 trials (6 approach angles x 3 shooting distances x 3 rounds) and lasted about 10 min.

#### Training Session

**Fig. 2:**
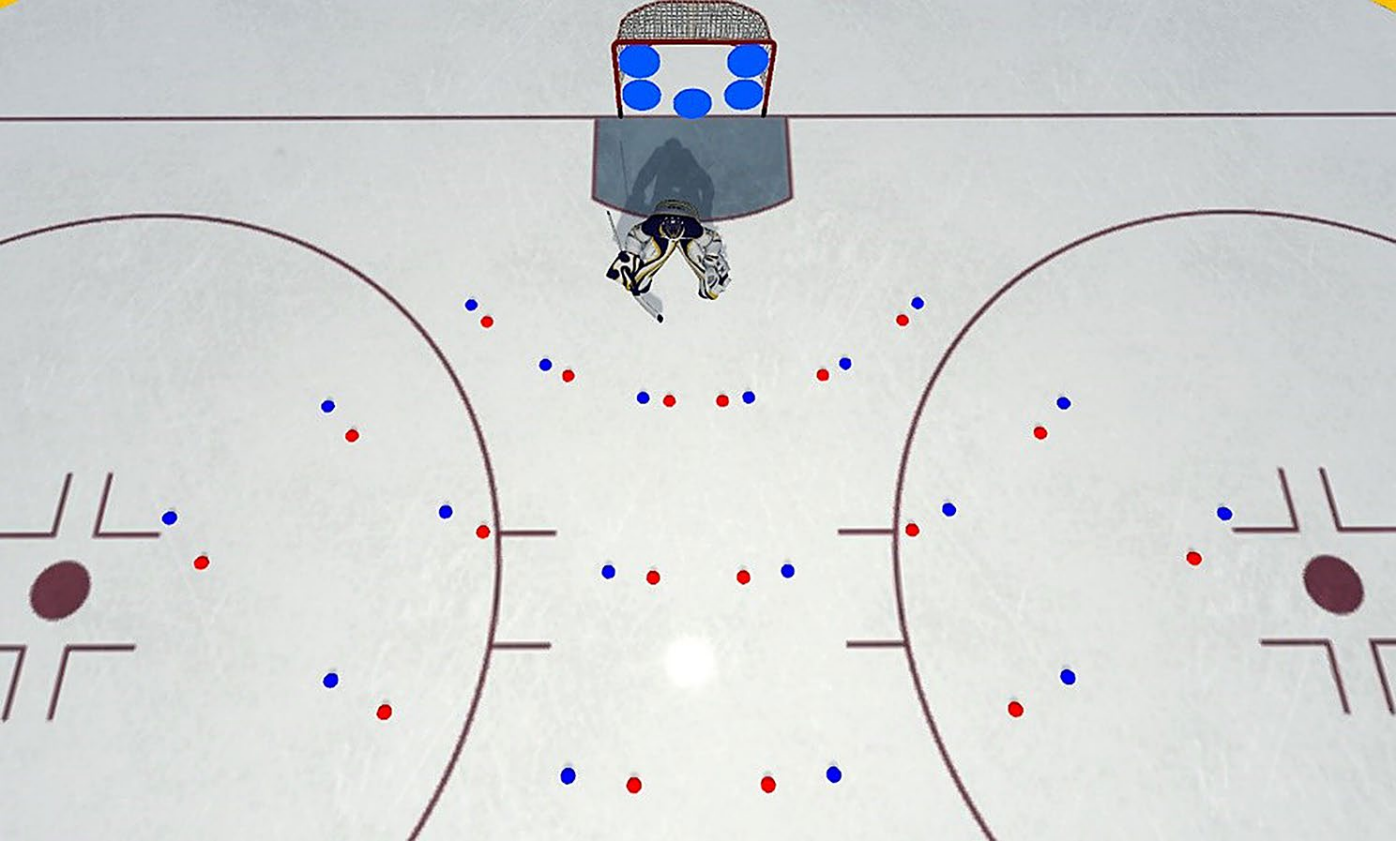
Shooting positions used in the test (i.e., pre- and post-, blue dots) and training sessions (red dots)

The training session closely mirrored the pre- and post-test sessions, with two key differences. First, upon selecting the least covered target area (i.e., LEA target) and pressing one of the response buttons, players in the feedback group were provided with a five-second feedback regarding the actual LEA target from the puck perspective, while players in the control group observed the goal and target for five seconds. Consequently, training trials lasted five seconds longer than pre- and post-test trials. Additionally, the approach angles in the training session were different from those used in the test sessions. Specifically, to mitigate potential learning biases, all angles were shifted by 5°, so that the approach angles in the training session were: 50°, 30°, 10°, -10°, -30°, and − 50°. However, the three shooting distances were the same as in the test sessions, namely 3.5, 5.5, and 7.5 m. The rationale for employing different approach angles in the training and test sessions was to prevent players from simply memorizing the LEA for specific shooting locations. Instead, this approach aimed to reinforce the learning of a broader ‘rule’ or general relationship between visual information and the LEA from the puck perspective. Figure 2 provides an overview of the shooting angles and distances used across the various sessions. Each shooting location featured three randomly presented training trials, again resulting in a total of 54 trials. The training session lasted about 15 min.

#### Measured Cognitive Ability (Dependent Variable)

The dependent variable was the perceptual ability to visually estimate the LEA, i.e., the ‘best’ target in terms of free space. This ability was measured using a score ranging from 0 to 1. For each trial, the score 1 was obtained when the target selected by the player corresponded to the LEA from the puck perspective, i.e., the target with the largest surface of ‘free space’ for the puck to reach the net. The score attributed to each of the other targets depended on their surface of ‘free space’ proportionally compared to the LEA target. Specifically, we created five raytracing cones starting from the puck shooting position and pointing to the five circular target areas. Each circular area was the base of a raytracing cone, which was itself composed of 121 rays evenly distributed in the volume. The chosen number of rays was a tradeoff between the precision of the discretization process and the computing time. Hence, 605 rays (5 × 121) were traced from the puck position to their dedicated positions in the circular target areas. Rays intersecting the goaltender virtual mesh (i.e., its shape) were intercepted (Supplemental Fig. 4 illustrates the procedure), whereas others could reach their target. The circular area receiving the maximum number of rays was elected as the LEA target. Dividing the number of rays received by each circular area by the number of rays received by the LEA target (i.e., the target receiving the largest number of rays) allowed us to give normalized scores ranging from 0 to 1.

#### Feedback

**Fig. 3:**
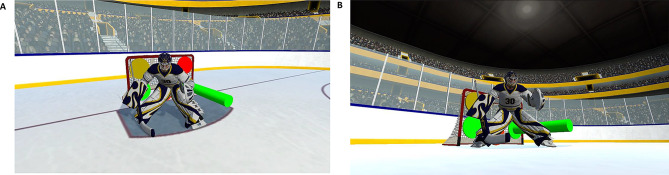
The two types of feedback provided to the feedback group during the training session. **A**. Eyes-view with color-coding and protruding targets. **B**. Puck-view

The feedback (feedback group only) consisted of indicating to the players which target was the least covered target from the puck perspective (i.e., LEA). At the end of each training trial, two types of feedback were consecutively presented, always in the same order. The first type of feedback relied on a combination of color-coding and extrusion. Specifically, the LEA target was highlighted in green, whereas the most covered target was highlighted in red. For the other targets, the color spectrum smoothly transitioned between red and green, visually representing intermediate selections. In addition to the color coding, the LEA target was also visually emphasized through a three-dimensional extrusion, namely a protruding cylinder whose base was the target. Note that for this type of feedback, the perspective on the scene remained unchanged, i.e., it was a first-person-view. Figure 3A shows an example of this type of feedback. The second type of feedback consisted of changing the camera perspective to show the scene (i.e., goal, goaltender and target areas) from a puck perspective (see Fig. 3B), thereby granting a unique vantage point on the visual scene. The two types of feedback were presented consecutively, each for 2.5 s, for a total feedback duration of 5 s. The participants in the control group did not receive any feedback and simply observed the goal for 5 s.

### Statistical Analysis

We first evaluated the baseline for perceptual performance, graded on a scale from 0 to 1. For each group (i.e., control and feedback) analyzed separately, we compared the average performance during the pre-test session with the maximum score of 1 using the Wilcoxon signed rank exact test (i.e., a one-sample test). We also directly compared the mean pre-test scores between the two groups. This comparison allowed us to control that the baseline score was similar in the two groups in order to rule out the possibility that training-evoked differences could partially result from initial differences between groups (even though participants were randomly assigned to the different groups). We then assessed whether the training session gave rise to a significant improvement of the baseline score. Specifically, for each group, we compared the mean score obtained after training (post-test session) with the mean score obtained before training (pre-test session). We also directly compared the training-evoked improvement between the two groups. Specifically, for each participant, we computed the difference between the score obtained in the post-test session and the score obtained in the pre-test session. We then compared the mean improvement between groups. To complete the analysis, we compared the mean score after training between groups.

Pre- vs. post- comparisons within groups were performed using Wilcoxon signed rank tests for repeated measures, whereas between groups comparisons were performed using Wilcoxon rank sum tests. For each comparison, the Bayes factor, i.e., the ratio between the likelihood of competing hypotheses, was computed to estimate the likelihood that the null vs. the alternative hypothesis was true. The computed Bayes factor was interpreted following the suggestions of Andraszewicz and colleagues [97]. Pearson’s R was computed as an indicator of the effect size.

We also assessed whether and to what extent the score (i.e., the perceptual performance) was related to the eyes-puck difference, namely the difference between the LEA target from a puck perspective and the LEA target from the eyes perspective. Specifically, the eyes-puck difference was calculated as the difference between the ray trace coverage of the LEA target from the puck viewpoint and the LEA target from the eyes viewpoint, resulting in a value ranging from − 1 to 1. The strength of the relationship between the eyes-puck difference and the score was quantified using a correlation coefficient. We also fitted a linear model to the data and computed the goodness of fit using the adjusted R-squared. This was done separately for the data collected in the pre- and post- sessions. Note that the data collected in the control group were not included in this analysis because there was no significant effect of the training session for this group (i.e., no significant difference between the pre- and the post- session, see Results section below). Note also that in order to choose a correlation coefficient, namely either the Pearson’s R or the Spearman’s rho, we extracted the residuals of the linear model fitted to the data, and we assessed whether they were normally distributed using the Shapiro-Wilk test.

Then, because two separate linear models were applied to the pre-test and post-test data, we assessed whether the slopes of the two models were statistically different from one another. For that, we first fitted a new linear model to the data. This new model included the session as predictor, as well as an interaction term between the eyes-puck difference and the session. We used a robust linear model (non-parametric) because the residuals of the linear model were not normally distributed.

Finally, we completed our analysis by modeling the results of the experiment using a Bayesian model. We first clustered the type of trials into two classes, namely good trials (whose scores are close to 1) and bad trials (whose score are significantly below one). The justification of this clustering is twofold: first, due to the method used to measure scores (i.e., casting of a large collection of randomly directed rays, see Methods section), the measured value of the score is itself subject to small errors, and a target with a score of 0.99 might actually be better than, or at least as good as a target with a score of 1; and second, scores close to 1 are extremely likely to be valid targets in practice. After clustering the trials into good and bad trials, a Bayesian logistic regression was performed for each player and each test session (i.e., pre- and post-). This regression predicted the chance of picking a good target as a function of the eyes-puck difference. We chose a uniform prior on the interval [-5,5] for the intercept of the regression, and a uniform prior on the interval [-5,0] for the slope. In this model, the slope of the logistic regression represents the impact of the eyes-puck difference, so that smaller slope values indicate a better ability to integrate the difference of perspective between the eyes and the puck. We then compared the distribution of the slopes of the logistic regression for each group (control / feedback) and for both test sessions (pre-/post-).

## Results

### Baseline for Perceptual Performance (Pre-Test Session)

For both groups, the average baseline score (0.76 ± 0.05 and 0.78 ± 0.07 for the control and feedback group, respectively) was significantly lower than 1 (*p* <.001 and *R*=-.88 in both cases). In addition, the baseline score was not different between the two groups (i.e., 0.76 vs. 0.78, *p* =.54, *R* =.11). This was confirmed by the Bayes factor (BF = 0.38), which indicated anecdotal evidence in favor of the null hypothesis, i.e., no difference between the two groups. The average baseline scores of the two groups of professional players were then compared to the average score of seventeen age-matched amateur players who performed the same task (i.e., pre-test session, see Supplementary information for details). This comparison was performed to ‘validate’ the relevance of the perceptual ability to identify the LEA from a puck perspective as a marker of perceptual expertise in ice hockey. Specifically, Ericsson and colleagues [98] defined expertise as “…the characteristics, skills, and knowledge that distinguish experts from novices and less experienced people”. Similarly, Marteniuk [99] proposed that perceptual skills refer to “…the ability to identify and acquire environmental information for integration with existing knowledge such that appropriate responses can be selected and executed”. Accordingly, we reasoned that if being able to identify the LEA from a puck perspective is a relevant perceptual skill in ice hockey, then professional players should score higher in our task than amateur players. As shown in Supplemental Fig. 5, the average scores of the two groups of professional players (see values above) were significantly higher (*p* <.001) than the average score measured for the group of amateur players (mean = 0.64 ± 0.08). This result suggests that the ability to identify the LEA from a puck perspective is indeed a relevant marker of perceptual expertise in ice hockey (though obviously not the only one).

### Training Effect for Each Group: Post-Test Vs. Pre-Test Comparison

**Fig. 4:**
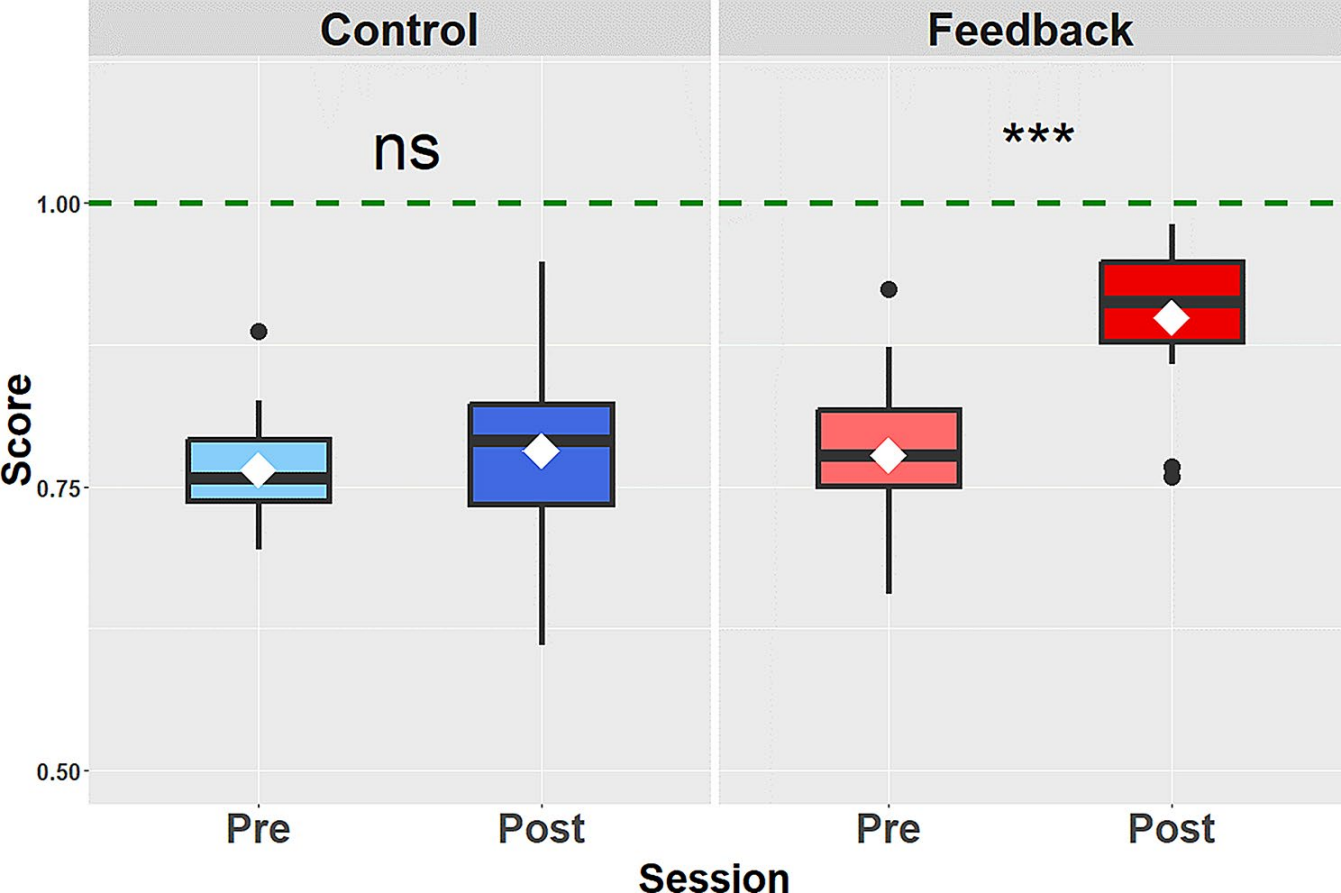
Perceptual performance (score from 0 to 1) for the two groups in the two test sessions (pre- and post-). The perceptual performance was significantly better after training (post-test session) for the feedback group (red) but not for the control group (blue). The black filled circles indicate outliers. The triple asterisk indicates a p-value < 0.001, and ‘ns’ means that the difference was not significant

For the control group, i.e., when no feedback was provided during the training session, the estimation performance was slightly better in the post-test (mean = 0.78 ± 0.08) than in the pre-test session (mean = 0.76 ± 0.05), but this difference was not significant (*p* =.21, *R* =.32). The computed Bayes factor was 0.39, which constitutes anecdotal evidence in favor of the null hypothesis, i.e., no difference between the post- and pre-test sessions. Additionally, we also ran a power analysis (with power set at 0.9 and alpha set at 0.05) which indicated that a minimum of 170 participants would be required to possibly detect a significant difference between post- and pre- for this group. On the other hand, when feedback was provided during the training session, the perceptual performance was significantly better in the post-test (mean = 0.90 ± 0.06) than in the pre-test session (mean = 0.78 ± 0.07; *p* <.001, *R* =.87). The computed Bayes factor was 94,413, which constitutes extreme evidence in favor of the alternative hypothesis, i.e., a real difference between the post- and pre-test sessions. Figure 4 shows the scores obtained in the two groups before (pre-) and after (post-) training.

### Between Groups Comparison of the Training Effect

**Fig. 5:**
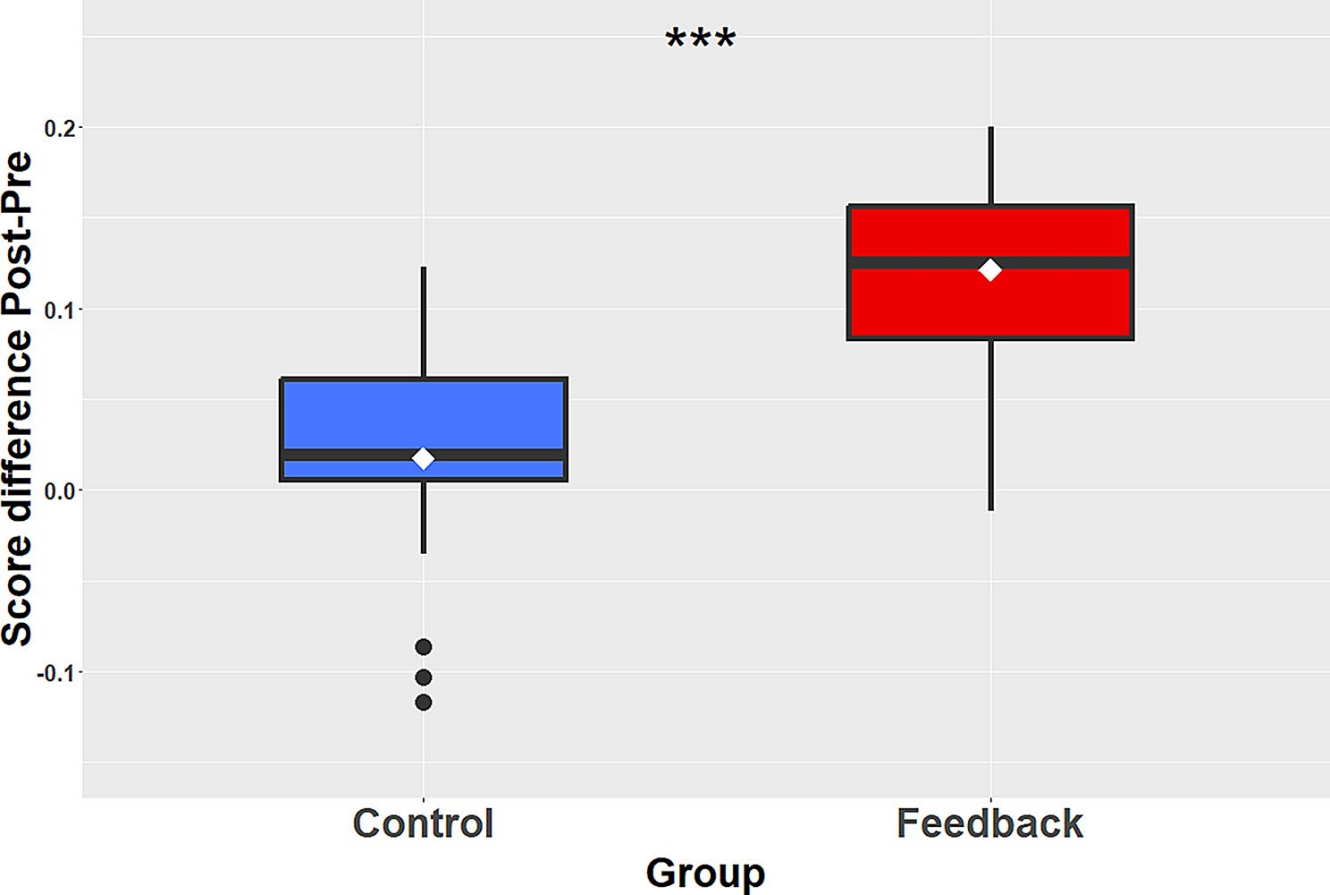
Average score improvement for the two groups. The improvement was significantly larger for the feedback (red) than for the control group (blue). The black filled circles indicate outliers, and the triple asterisk indicates a p-value < 0.001

When directly comparing the effect of training between the two groups, we observed that the mean improvement was significantly larger for the feedback (mean = 0.12 ± 0.06) than for the control group (mean = 0.02 ± 0.07; *p* <.001, *R* =.65). The Bayes factor was 421, which indicates extreme evidence in favor of the alternative hypothesis, i.e., a real difference between the two groups. Figure 5 shows the score improvement after training for the two groups.

In line with the previous result, the mean scores measured in the post-test session were significantly larger for the feedback group than for the control group (0.90 ± 0.06 vs. 0.78 ± 0.08; *p* <.001, *R* =.61). This was confirmed by the Bayes factor (BF = 329), which indicated extreme evidence in favor of a real difference between the two groups. Note however that despite the score improvement measured for the feedback group, the score in the post-test session was still significantly lower than the maximum score of 1 for both groups (*p* <.001 in both cases, as assessed using Wilcoxon signed rank exact tests).

### Relation between the Eyes-Puck Difference and the Score (Estimation Performance)

**Fig. 6:**
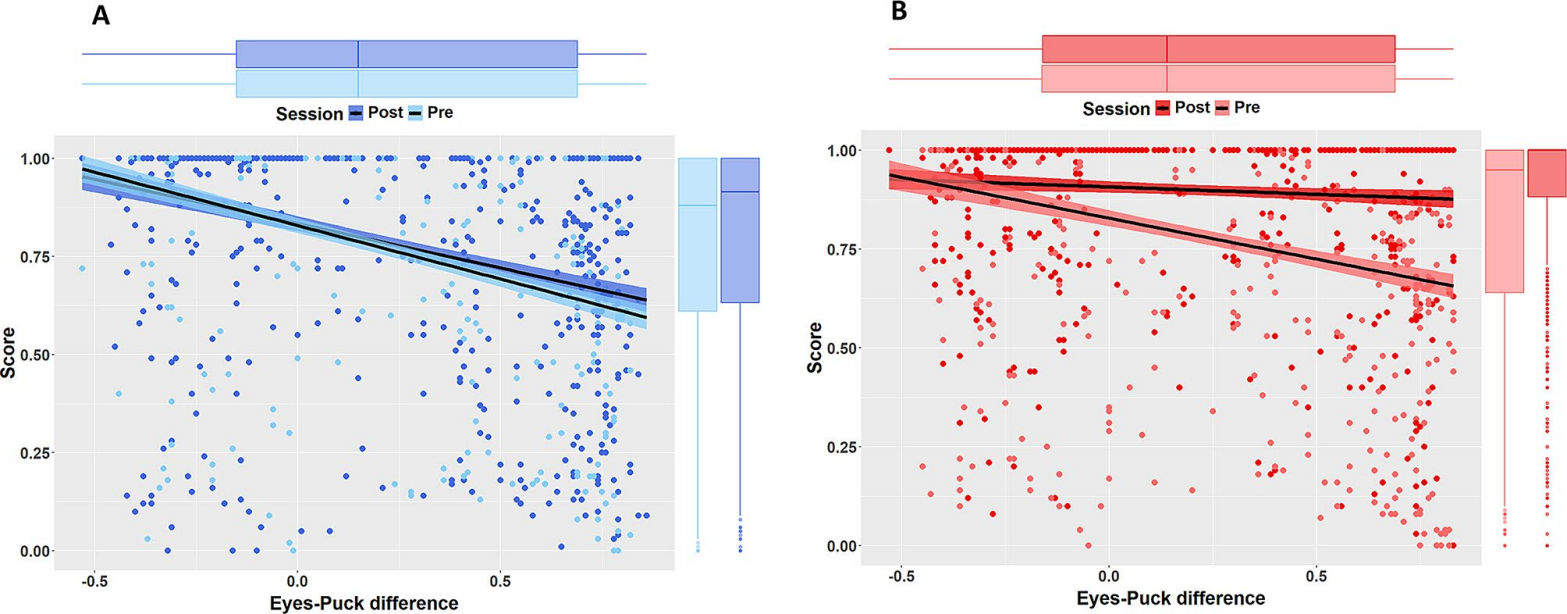
Relationship between the eyes-puck difference and the estimation performance. The relationship is shown for the two test sessions (pre- in light color, post- in darker color). **A**. Control group (blue). The effect of the eyes-puck difference on the perceptual performance is similar before and after training. **B**. Feedback group (red). Training strongly reduced the effect of the eyes-puck difference on the perceptual performance

Figure 6 shows the relationship between the eyes-puck difference and the score for the control (6A) and the feedback group (6B).

#### Control Group

For the pre-test session, the measured Spearman’s rho was − 0.40, which indicates a weak, negative correlation between the eyes-puck difference and the score. Note that we used the Spearman’s rho rather than the Pearson’s R because the residuals of the linear model were not normally distributed. The equation of the regression line was: Y=-0.26X + 0.82 (-0.27X + 0.87 using a robust regression), and the slope was significantly different from 0 (*p* <.001). The adjusted R-squared was 0.14, indicating that the eyes-puck difference explained 14% of the variance of the score.

Regarding the post-test session, the measured Spearman’s rho was − 0.37, indicating a weak negative correlation between the two variables. The equation of the regression line was: Y=-0.23X + 0.83 (-0.24X + 0.89 using a robust regression), and here again, the slope was significantly different from 0 (*p* <.001). The adjusted R-squared was 0.12, meaning that the eyes-puck difference explained 12% of the variance of the score.

A visual comparison of pre- and post-test data (Fig. 6A, light blue vs. dark blue dots) and an inspection of the slope values measured with the two linear models suggested that the effect of the eyes-puck difference on the score was similar for the pre- and post-test sessions, which would indicate that the training did not alter the relationship between the two variables. We fitted a new linear model to the data to assess whether the slopes of the two linear models (pre- vs. post-) were statistically different from one another. This new linear model included the session as predictor, as well as an interaction term between the eyes-puck difference and the session. The results indicated no significant interaction between the eyes-puck difference and the session (F(1, 1831) = 2.57, *p* >.05), confirming that there was no significant difference between the two slopes. In other words, for the control group, who did not receive feedback during the training session, the influence of the eyes-puck difference on the perceptual performance was the same before and after training.

#### Feedback Group

For the pre-test session, the measured Spearman’s rho was − 0.29, which indicates a weak, negative correlation between the eyes-puck difference and the Score. Here again, we used the Spearman’s rho because the residuals of the linear model were not normally distributed. The equation of the regression line was: Y=-0.18X + 0.82 (-0.17X + 0.88 using a robust regression), and the slope was significantly different from 0 (*p* <.001). The adjusted R-squared was 0.06, indicating that the eyes-puck difference explained only 6% of the variance of the score.

Regarding the post- session, the measured Spearman’s rho was − 0.07, indicating no correlation between the two variables. The equation of the regression line was: Y=-0.04X + 0.91 (-0.00X + 0.99 using a robust regression), and here again, the slope was significantly different from 0 (*p* <.05). The adjusted R-squared was 0.01, meaning that the eyes-puck difference only explained 1% of the variance of the score.

A visual comparison of pre-test and post-test data (Fig. 6B, light red vs. dark red dots) and an inspection of the slope values measured with the two linear models suggested that the effect of the eyes-puck difference on the score was different for the post- and pre-test sessions. Specifically, in the pre-test session, higher eyes-puck differences tended to lead to lower scores / estimation performance (Fig. 6B, light red dots). This was no longer the case in the post-test session (Fig. 6B, dark red dots). As for the control group, we fitted a new linear model to the data to assess whether the slopes of the two linear models (pre- vs. post-) were statistically different from one another. This new linear model included the session as predictor, as well as an interaction term between the eyes-puck difference and the session. The results indicated a significant interaction between the eyes-puck difference and the session (F(1, 1830) = 41.60, *p* <.001), confirming a significant difference between the two slopes. In other words, providing feedback during the training session strongly reduced the influence of the eyes-puck difference on the perceptual performance.

### Bayesian Analysis

#### Distribution of SCORES and Clustering

**Fig. 7:**
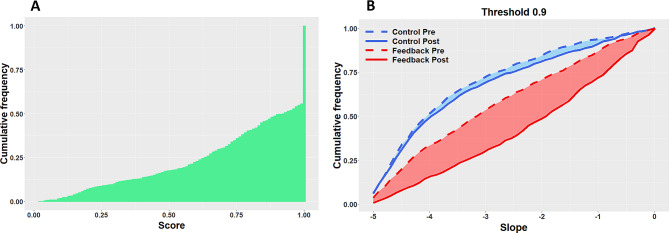
**A**. Cumulative distribution frequency of the scores observed throughout the experiment. **B**. Cumulative distribution frequency of slopes as computed with a Bayesian logistic regression, for a clustering with a threshold at 0.9. The figure shows the distribution for the two groups (control in blue and feedback in red) and the two test sessions (dashed lines for pre- and solid lines for post-). The closer to zero the slope is, the better is the perceptual performance to assess the LEA target from the puck perspective. Therefore, for each distribution, the smaller the area under the curve, the better the perceptual performance. One can clearly see a substantial score improvement after training for the feedback group, and this even though the baseline score was better (though non-significantly) in this group

As shown in Fig. 7A, the large majority of the trials had scores very close to 1, and more than 55% of the trials had a score above 0.99. This observation makes sense considering that all tested participants were professional players. Additionally, the distribution demonstrates strong smoothness below 0.99, which indicates that the choice of the limit between scores that are deemed ‘good’ and ‘bad’ is permissive. This means that thresholds of 0.9 vs 0.95 lead to very similar clusters. We set the threshold at 0.9 and labeled trials with a better score as good trials (vs bad trials for scores below the threshold).

#### Distribution of Slopes

Figure 7B shows the cumulative distribution frequency of slopes as computed with the Bayesian logistic regression with a clustering threshold at 0.9. For the control group, the probability of improvement after training is 0.501, with an average expected improvement of 0.07. Note that probabilities of improvement below 0.5 indicate that observing performance deterioration is more likely than observing performance improvement. Regarding the feedback group, the probability of improvement after training is 0.7, and the average expected improvement is 0.83. This latter value corresponds to a slope increase of 0.83, which means that it gets closer to zero (i.e., better perceptual performance). When setting a threshold at 0.95, the pattern of results is very similar. Specifically, the probability of improvement is 0.501 for the control group vs. 0.71 for the feedback group, and the average expected improvement is 0.071 for the control group vs. 0.82 for the feedback group.

## Discussion

We assessed the perceptual ability of professional ice hockey players to identify the LEA from a puck perspective. In addition, we tested whether a specific visual feedback showing the goal as would be seen from the puck could improve the perceptual performance.

For both groups of professional players, the average score before training was significantly better than that of amateur players (see Supplemental Information), but still significantly lower than 1, the maximum score. In other words, even though all participants were professional players, their initial perceptual performance was suboptimal. This indicates that the perceptual ability to ‘put oneself in the eyes of the puck’ is difficult to acquire, even after years of intensive practice at the highest level. Importantly, only one session of 15 min (54 trials) with our VR simulator and the provided feedback significantly improved the players’ perceptual performance. In particular, the scores in the feedback group were significantly higher after training, and this improvement was feedback-specific, as no significant improvement was measured for the control group. The feedback-specific nature of the measured improvement in perceptual performance was further substantiated by the between-groups comparisons, which showed that both the post-vs-pre-difference and the raw perceptual performance in the post-test session were significantly larger for the feedback group as compared to the control group. Note that these between-groups differences cannot be explained by a putative initial difference between the groups, as baseline score was similar for the two groups (i.e., no significant difference between the two groups in the pre-test session). Taken together, our results support the effectiveness of the feedback provided to the feedback group during training to improve perceptual performance. Yet, the score in the post-test session was still lower than 1, and this for both groups. This indicates that even though the specific feedback effectively improved perceptual performance in the proposed task, there is still some capacity for improvement. This seems reasonable when considering that the players only had one training session of 54 trials. In line with this, future experiments will measure the feedback-evoked improvement resulting of repeated training sessions spanning over several weeks.

Unsurprisingly, the initial perceptual performance (i.e., before training) was related to the amplitude of the difference between the eyes-view and the puck-view. Specifically, for both groups, larger eyes-puck differences were associated with lower scores, as evidenced by the slope of the regression line (see Fig. 6). For the control group, the training did not alter this trend, the score being similar before and after training. Conversely, for the players who received feedback during the training session (i.e., feedback group), the negative impact of the eyes-puck difference was significantly reduced after training. Specifically, the slope of the regression line was much closer to zero after training (see Fig. 6B). This shift was confirmed by the Bayesian analysis (see Fig. 7B). In other words, providing feedback during the training session led to the strongest perceptual improvement for large eyes-puck differences (see the regression lines pre- vs. post-), i.e., for angles / scenarios in which the initial score was the lowest. This seems logical because those scenarios were those with the ‘largest’ capacity for improvement. This point is interesting because even though the average baseline score was not significantly different between the two groups, the score of the feedback group was slightly higher, i.e., less affected by the eyes-puck difference (see Fig. 7B). In other words, the feedback group had in theory slightly less capacity for improvement than the control group. Yet, the training-evoked improvement was much larger for the feedback group than for the control group. This confirms the effectiveness of the feedback.

Importantly, the significant feedback-evoked improvement of perceptual performance reported here has been measured for professional players, i.e., players who already have extensive experience through many years of practice (see also [35]). This experience should have allowed them to integrate the difference between eyes- and puck-view. Yet, 15 min of training with our simulator improved their perceptual performance by 12%, which is rather substantial for professional athletes. Indeed, according to the well-known power law of practice [70, 100–102], performance improvement is rapid at first but grows systematically smaller as practice continues [73]. As highly trained experts, professional athletes are at the high-end of the practice/abilities continuum and should therefore be much less ‘susceptible’ / prone to sizeable improvement, especially over very short periods of training. In this context, the substantial improvement of perceptual performance measured after training in the feedback group likely reflects the unique nature of the training provided by our simulator. Specifically, giving players the opportunity to have a puck-view on the visual scene (in our specific scenario on the goal) would be difficult without the help of VR technology. The large improvement measured in our experiment after only 15 minutes of specific feedback-based training suggests that this perceptual skill is not easily acquired via regular training and extensive practice. In the near future, the rapid development of AR technology will likely allow trainers to propose similar training protocols directly on the ice rink, which will further increase the representativeness of the training situations and the transfer rate.

## Conclusion

In recent years, there has been a growing interest in the perceptual and cognitive abilities of athletes [18, 21]. In team sports, decision making is highly dependent on the position, movements and actions of both partners and opponents [25–27]. Specifically, whether in cooperative or competitive settings, athletes continuously monitor the actions of their partners and/or opponents in order to adapt their own motor actions to the current situation [103–106]. In this context, the use of human-avatar interaction and virtual opponents can constitute an excellent training tool [59, 82, 107, 108]. This is even truer when the interaction offers the possibility of training skills which could not be trained in the ‘real world’ / using traditional training methods. In line with this, our study is a good illustration of the way VR can be used to improve perceptual skills in team sports, even in highly trained professional athletes.

## Electronic supplementary material

Below is the link to the electronic supplementary material.

Supplementary Material 1

## Data Availability

Raw human experimental data will be deposited on Elsevier’s Mendeley Data Repository and be publicly available as of the date of publication.

## Abbreviations

VR: Virtual reality
AR: Augmented reality
LEA: Largest exposed area
