# Supplementary material for: Perceptual Training in Ice Hockey: Bridging the Eyes-Puck Gap Using Virtual Reality
Source: Sports Med Open. 2025 Apr 12;11:38. doi: 10.1186/s40798-025-00840-x (PMC11993527; doi:10.1186/s40798-025-00840-x)

**Supplemental Fig. 1.** Illustration of the motion-capture session underlying the animation of the virtual goaltender. A. The professional goaltender is equipped with infrared reflective markers and performs saves on artificial ice in our tracking lab (24 infrared cameras). B. The virtual goaltender is animated using the captured save movements of the real goaltender.

**Supplemental Fig. 2.** Illustration of the eyes-puck difference and of how it was measured.

**Supplemental Fig. 3.** The home-made response box used for the experiment. The configuration of the response buttons matched the configuration of the targets in the goal.

**Supplemental Fig. 4.** Illustration of the ray-casting technique used to identify the amount of ‘exposed’ area for each target.

**Supplemental Fig. 5**. Baseline score / perceptual performance (from 0 to 1) for the two groups of professional players (blue and red boxes) and the group of amateur players (green box). The score was significantly higher for the two groups of professional players than for the amateur players. The black filled circles indicate outliers. The triple asterisk indicates a p-value < .001, and ‘ns’ means that the difference was not significant.

**SUPPLEMENTARY INFORMATION**

**Baseline for perceptual performance: amateur vs professional players**

Seventeen amateur participants performed the pre-test session of the experiment, namely the same baseline test as the one performed by the professional players. The amateur group included thirteen male and four female participants, aged 19 to 34 (mean = 26.12). Eleven of them play left-handed and the other six play right-handed. All were sporting individuals whose main sport was not ice hockey but who practiced ice hockey as a leisure activity, i.e., not competitively. The average score of the amateur participants was 0.64 ± 0.08 (vs 0.76 ± 0.05 and 0.78 ± 0.07 for the two groups of professional players). Note that the scores for the female participants in the amateur group were 0.79, 0.77, 0.63 and 0.64, i.e., equal to or higher than the average score of the group.

We ran a Kruskal-Wallis test to compare the average score of the two groups of professional players in the pre- session (i.e., before training) and the average score of the group of amateur players. This test indicated a significant difference between the three means (χ2(2) = 20.63, p < .001, with a large effect size, namely an epsilon squared of 0.41). Bonferroni-corrected paired comparisons indicated no significant difference between the two groups of professional players (p = .54), whereas the average score of the amateur players was significantly lower than that of both groups of professional players (p < .001 in both cases, see Supplemental Fig. 5).

**SUPPLEMENTAL FIGURES**

**Supplemental Fig. 1**

**A**


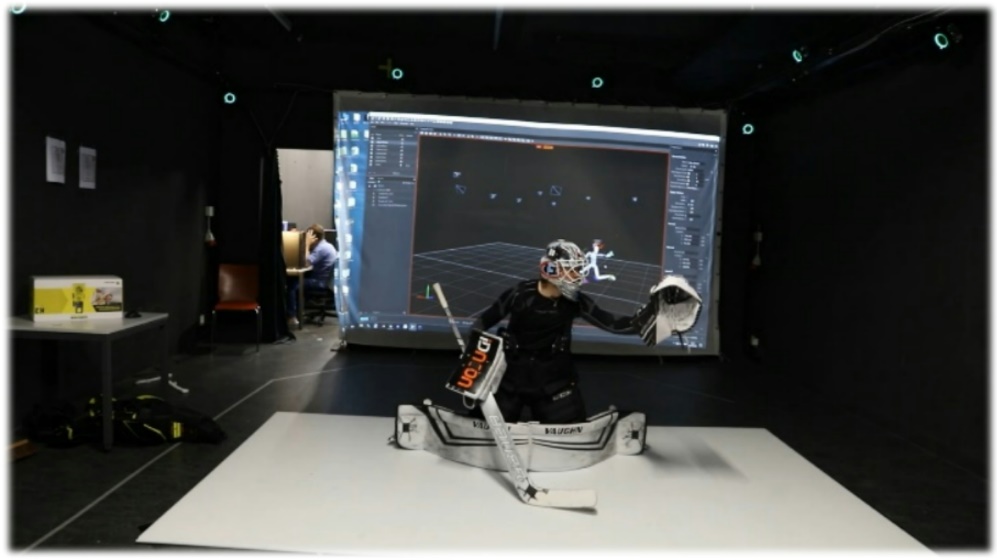


**B**


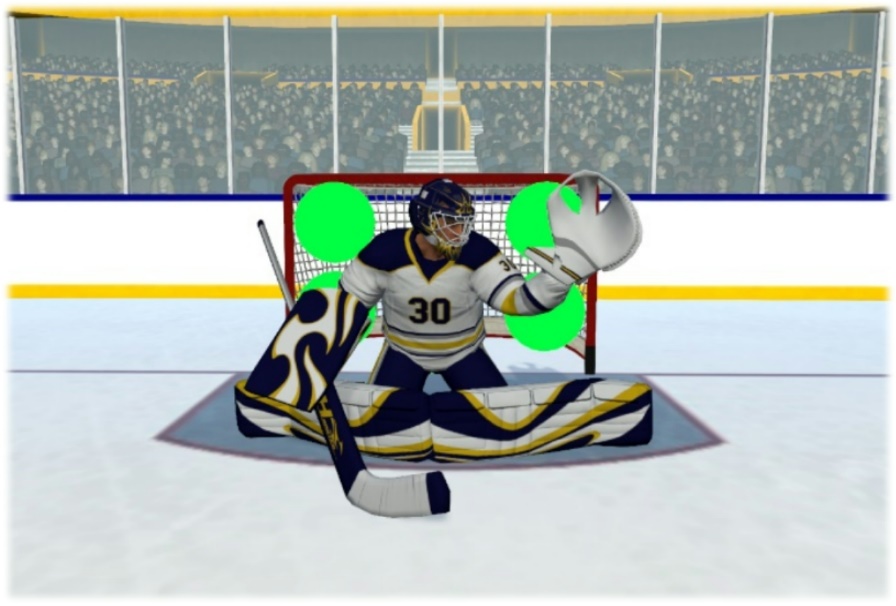


**Supplemental Fig. 2**


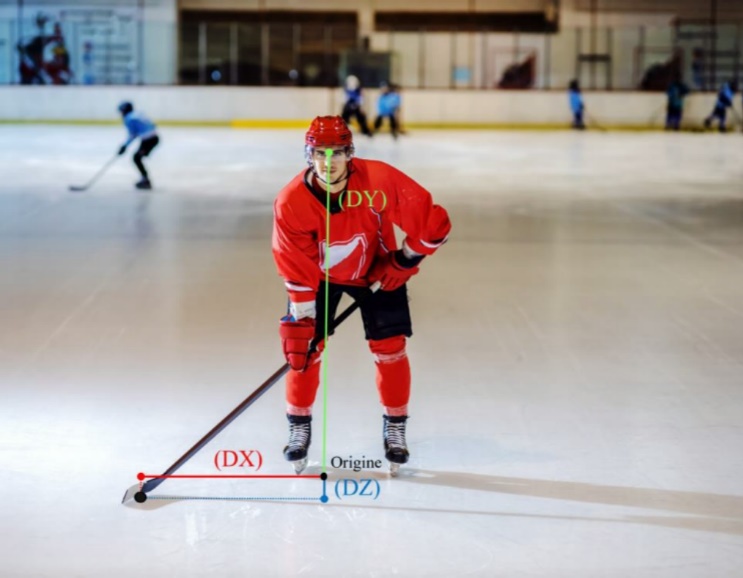


**Supplemental Fig. 3**


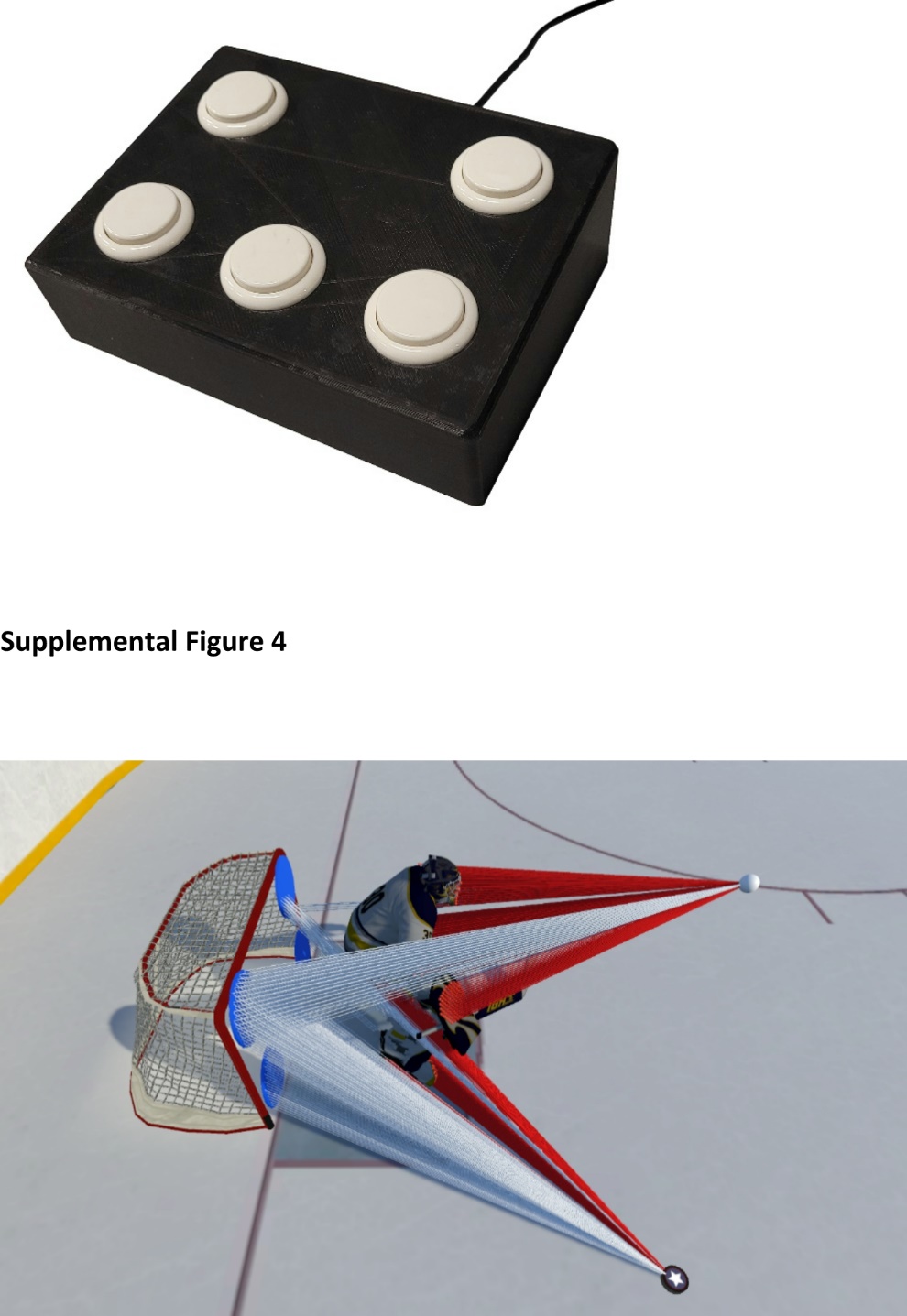


**Supplemental Fig. 5**


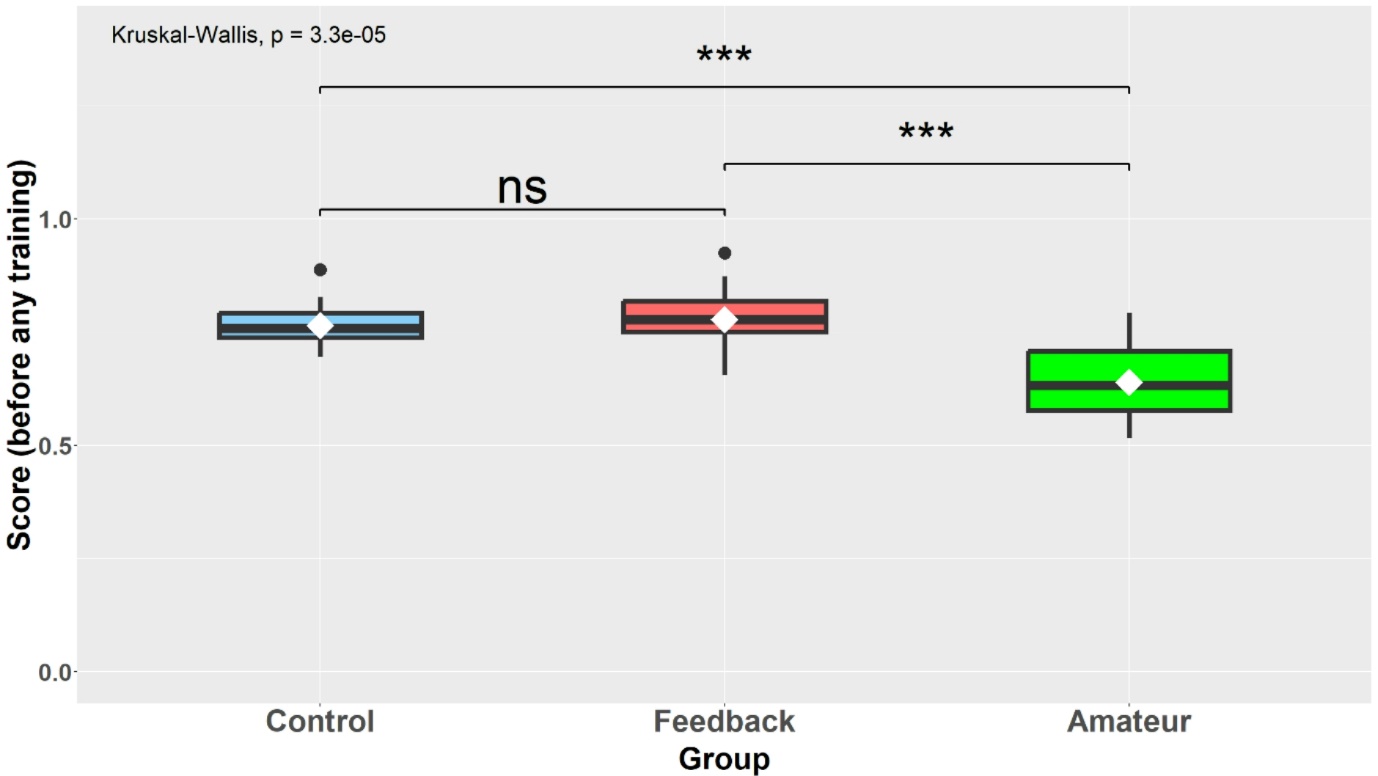

Supplement: Supplementary file 1 — Supplementary Material 1 [file 40798_2025_840_MOESM1_ESM.docx]
